# A Cost-Effective Nano-Sized Curcumin Delivery System with High Drug Loading Capacity Prepared via Flash Nanoprecipitation

**DOI:** 10.3390/nano11030734

**Published:** 2021-03-15

**Authors:** Zhuo Chen, Zhinan Fu, Li Li, Enguang Ma, Xuhong Guo

**Affiliations:** 1 State Key Laboratory of Chemical Engineering, East China University of Science and Technology, Shanghai 200237, China; chen9635@163.com; 2 Engineering Research Center of Materials Chemical Engineering of Xinjiang Bingtuan, Shihezi University, Shihezi 832000, China; maenguang@163.com

**Keywords:** flash nanoprecipitation (FNP), nanoparticle, amphiphilic homopolymer, chitosan

## Abstract

Flash nanoprecipitation (FNP) is an efficient technique for encapsulating drugs in particulate carriers assembled by amphiphilic polymers. In this study, a novel nanoparticular system of a model drug curcumin (CUR) based on FNP technique was developed by using cheap and commercially available amphiphilic poly(vinyl pyrrolidone) (PVP) as stabilizer and natural polymer chitosan (CS) as trapping agent. Using this strategy, high encapsulation efficiency (EE > 95%) and drug loading capacity (DLC > 40%) of CUR were achieved. The resulting CUR-loaded nanoparticles (NPs) showed a long-term stability (at least 2 months) and pH-responsive release behavior. This work offers a new strategy to prepare cost-effective drug-loaded NPs with high drug loading capacity and opens a unique opportunity for industrial scale-up.

## 1. Introduction

Cancer has become a major public problem worldwide, threatening human health seriously due to its increasing prevalence [1]. Although more and more anti-cancer drugs have been developed, many have low water solubility [2]. For instance, curcumin (CUR) has been demonstrated as an effective anti-cancer drug regulating almost all types of cancer signs, including cell proliferation, cancer-related inflammation, and cell apoptosis [3,4,5,6,7]. However, the low-solubility (<1.0 μg/mL) in aqueous solution of the naked CUR leads to inadequate body intake and the lower therapeutic efficacy [8]. Therefore, it is necessary not only to design novel drug delivery systems but also to propose efficient treatment strategies to improve the cure outcome. Polymeric nanoparticles (NPs) have been demonstrated as a promising opportunity to load hydrophobic anti-cancer drugs [9,10,11,12,13], in which amphiphilic polymers are commonly used as stabilizers to protect hydrophobic drugs through the intermolecular forces such as hydrogen bonding, hydrophobic, and electrostatic interactions, thus improving the solubility of hydrophobic drugs in aqueous solution and prolonging the internal circulation time [14,15].

Flash nanoprecipitation (FNP) has been developed as an efficient and simple approach for the controlled fabrication of drug-loaded NPs [16,17,18,19,20,21]. In FNP, amphiphilic stabilizers and hydrophobic active ingredients are molecularly dissolved in a water-miscible organic solvent and rapidly mixed with aqueous antisolvent streams to drive controlled precipitation of the solutes, leading to the formation of NPs with tunable particle sizes (from 30 nm to 2 μm) and high encapsulation efficiency (>90%) [22,23]. In most cases, amphiphilic block copolymers (BCPs) are commonly used as stabilizers in the FNP technique [16]. For instance, Chow’s group successfully stabilized hydrophobic drug CUR with poly(DL-lactide)-b-polyethylene glycol (PLA-b-PEG) via FNP approach followed by a freeze-drying process to generate stable CUR-loaded NPs [24,25]. Zhu and coworkers exploited a multi-inlet vortex mixer (MIVM) to encapsulate a common model drug β-carotene using various di-block copolymers, such as PLA-b-PEG, polystyrene-block-poly(ethylene glycol) (PS-b-PEG), polycaprolactone-block-poly(ethylene glycol) (PCL-b-PEG), and poly(lactic-co-glycolic acid)-block-poly(ethylene glycol) (PLGA-b-PEG) during the FNP process and achieved a high drug loading capacity of 83% [26]. However, the high cost of these synthetic BCPs is still obstructive for their industrial scale-up.

In this work, our interest lies in the use of a cost-effective alternative as a stabilizer and hydroxyl-rich chitosan (CS) as a drug trapping agent to prepare drug-loaded NPs with higher drug loading capacity via FNP. Upon using the CS as a drug trapping agent, the resulting CS-containing CUR-loaded NPs with high drug encapsulation efficiency (>95%), excellent stability (>2 months), and pH-responsive drug release ability can be easily obtained, as compared to that of CS-free CUR-loaded NPs. Furthermore, the particle sizes of CUR-loaded NPs can be easily controlled by tuning mixing Reynolds number (Re). This study provides important insights for the cost-effective, rapid, and scalable preparation of drug-loaded NPs through the FNP technique.

## 2. Materials and Methods

### 2.1. Materials

Curcumin (CUR, 98.0%) was purchased from Adamas-beta (Shanghai, China). Poly(vinyl pyrrolidone) K-30 (PVP, ≥ 95.0%) was purchased from Solarbio science & technology Co., Ltd (Beijing, China). Chitosan (CS) with low molecular weight was purchased from Sigma-Aldrich (Shanghai, China). Sodium dodecyl sulfate (SDS, 99.0%) was purchased from J&K Chemicals. Acetic acid (99.0%) was purchased from Alladin (Shanghai, China). Sodium dihydrogen phosphate (NaH_2_PO_4_), sodium phosphate dibasic (Na_2_HPO_4_·12 H_2_O), tetrahydrofuran (THF), dichloromethane (DCM), and methyl alcohol were purchased from General-reagent (Shanghai, China). Deionized water was obtained from a Milli-Q water purification system and used in all experiments. All materials were used without further purification.

### 2.2. Preparation of CUR-loaded NPs

CS-containing CUR-loaded NPs were produced using the antisolvent principle by flash nanoprecipitation (FNP) via a multi-inlet vortex mixer (MIVM). Four inlets were connected to four hermetic stainless syringes via Teflon tubing. Two of the syringes were deionized water of 50 mL, the third one was CUR with CS in organic solvent tetrahydrofuran (THF) at the concentration of 3 mg/mL and 0.125 mg/mL, respectively, and the fourth one was PVP in water at the concentration of 6 mg/mL. The ratio of injection speeds of the streams was digitally controlled by syringe pumps PHD2000 (Harvard Apparatus Inc, Holliston, MA, USA).

The organic solvent with unencapsulated CUR was removed from nanosuspension by dialysis. The samples were sealed inside a dialysis bag (molecular mass cutoff with 10 kDa) and dialyzed against deionized water (600 mL of deionized water per 10 mL of NPs suspension) stirred at 500 rpm at ambient temperature for 24 h. The water was changed every 6 h. To facilitate subsequent characterization, the prepared nanosuspension was freeze-dried.

Reynolds number (Re) is a typical controllable kinetic parameter in FNP technology. Liu pointed out that in MIVM geometry, mixing was characterized under various inlet configurations and demonstrated homogeneous mixing conditions for mixing at Re > 1600 [27]. The definition of the Re is as follows:

Re =∑i=1nRei=∑i=1nρiQidsµi

where d is the stream inlet diameter of the mixer (1.1 × 10^−3^ m), s devotes the cross-sectional area of the inlet (1.65 × 10^−6^ m^2^), and the four inlets have the same d and s; ρ_i_ is the fluid density (kg/m^3^), μ_i_ is the fluid viscosity (Pa·s), and Q_i_ is the steam flow rate (m^3^/s). At 20 °C, ρ_water_ is 1.0 × 10^3^ kg/m^3^, and ρ_THF_ is 8.89 × 10^2^ kg/m^3^; μ is 5.5 × 10^−4^ Pa·s for THF and 1.0 × 10^−3^ Pa·s for water.

### 2.3. Encapsulation Efficiency and Drug Loading Capacity

The encapsulation efficiency (EE) and drug loading capacity (DLC) of the CUR-loaded NPs were determined using an ultraviolet-visible spectrophotometer. A certain amount of nanosuspension after dialysis was dissolved in a mixed solution of dichloromethane and methanol with the volume ratio of dichloromethane to methanol of 3:1. The concentration of CUR in organic solvent was measured by ultraviolet-visible spectrophotometer UV-2550 (Shimadzu Inc., Shanghai, China), and the characteristic peak of absorption in CUR located at 423 nm. The encapsulated CUR concentration was determined from the linear regression of the absorbance versus the concentration of CUR. The concentration of CUR in NPs can be calculated by comparing the CUR standard curve in dichloromethane methanol solution. The EE and DLC were calculated by the equations as follows:

$$\mathrm{EE}\;(\%)\;=\;\frac{\mathrm{Total}\;\mathrm{amount}\;\mathrm{of}\;\mathrm{loaded}\;\mathrm{CUR}}{\mathrm{Total}\;\mathrm{amount}\;\mathrm{of}\;\mathrm{CUR}\;\mathrm{added}}\;\times \;100$$

$$\mathrm{DLC}\;(\%)\;=\;\frac{\mathrm{Total}\;\mathrm{mass}\;\mathrm{of}\;\mathrm{loaded}\;\mathrm{CUR}}{\mathrm{Total}\;\mathrm{mass}\;\mathrm{of}\;\mathrm{NPs}}\;\times \;100$$

### 2.4. Characterization

The average hydrodynamic diameter and polydispersity index (PDI) and zeta potential of the NPs were measured by dynamic light scattering (DLS) performed on a NICOMP 380 ZLS instrument (PSS Inc, Santa Barbara, CA, USA) with a scattering angle of 90°. Each measurement was repeated three times and the average was taken. Transmission electron microscopy (TEM, JEM-1200EX, Tokyo, Japan) was employed to observe the morphologies, internal structures, and size of CUR-loaded NPs. TEM grids (carbon type-B, 300 coper mesh) were purchased from Beijing Zhongjingkeyi Technology Co., Ltd (Beijing, China). A drop of nanosuspension was placed on the TEM grid and the water of the droplet was removed after retention 15–20 min before the observation. A UV-2550 UV–vis spectrophotometer was used to record the UV-vis absorption spectra of the NPs from 300 to 500 nm. Each measurement was repeated three times. X-ray diffraction (XRD) patterns were acquired on a Bruker D8 X-ray diffractometer (Bruker AXS Inc, Karlsruhe, Germany) with a Cu Kα radiation. The diffraction patterns were recorded from 3° to 50° at a scanning rate of 0.02°/s. Differential scanning calorimetry (DSC) was implemented by a TA2000 Instruments Discovery DSC (TA Inc, New Castle, DE, USA) to characterize the thermal properties of CUR, PVP, CS, and NPs. Samples (about 5–10 mg) were sealed in aluminum pans. Subsequently, the pans were heated from 30 to 270 °C at 10 °C/min. Infrared spectra were recorded through the conventional KBr (Merck, Darmstadt, Germany) technique by use of a PerkinElmer Spectrum 400 FT-IR spectrometer, with a resolution of 2 cm^−1^ and 30 scans in the range of 400–4000 cm^−1^.

### 2.5. Stability Test

The stability of NPs (after dialysis) was characterized by the change in particle size and size distribution (PDI) at room temperature under the sealed environment for two months. The encapsulation efficiency of the NPs was also measured before and after two months.

### 2.6. In Vitro Release

To study the release behavior of CUR-loaded NPs, 3 mL CUR suspension were transferred into a dialysis bag containing 27 mL of 0.01 M phosphate buffer solution (PBS) with different pH values 7.4, 6.5, and 5.5) in a centrifuge tube, which was shaken at a speed of 200 rpm/min at 37 °C. Meanwhile, 0.135 g SDS was added into the PBS aiming to increase the solubility of the CUR in PBS. At specific time intervals, 1 mL of solution outside the dialysis bag was taken out for analysis of CUR concentration and supplemented with an equal amount of fresh PBS.

## 3. Results

**Scheme 1:**
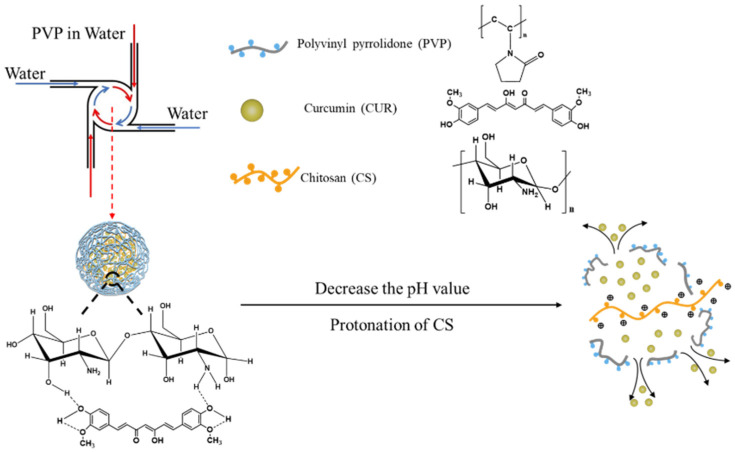
Schematic illustration of the preparation of the pH-responsive chitosan (CS)-containing curcumin (CUR)-loaded nanoparticles (NPs) by the flash nanoprecipitation (FNP) technique for the controlled drug delivery.

As shown in Scheme 1, nanoparticular system of curcumin (CUR) was prepared by FNP technique using poly(vinyl pyrrolidone) (PVP) and chitosan as additives. PVP works as the stabilizer for drug particles, while the natural polymer CS with good biocompatibility seems to be functionalized as a drug trapping agent in the FNP process to accelerate the nucleation of hydrophobic drugs leading to the high drug loading capacity. Moreover, the release behavior of drug within CS-containing drug-loaded NPs could be controlled by changing of pH at the targeted tumors and inflammation sites due to the protonation of amino groups in CS under acidic environment (Scheme 1).

### 3.1. Structural Characterization of CUR-Loaded NPs

**Figure 1:**
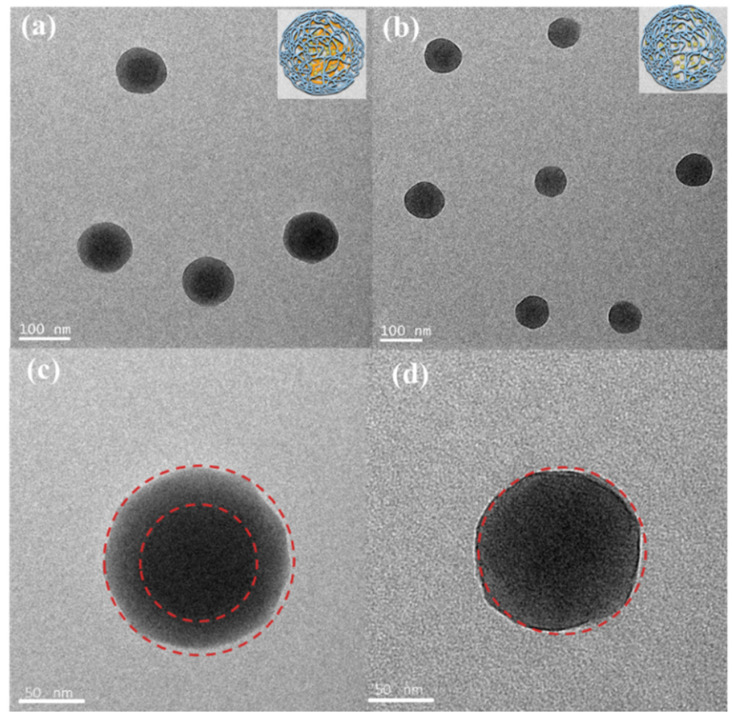
TEM images of (**a**) CS-containing and (**b**) CS-free CUR-loaded NPs, and (**c**,**d**) corresponding high-magnification TEM images of (**a**,**b**).

**Figure 2:**
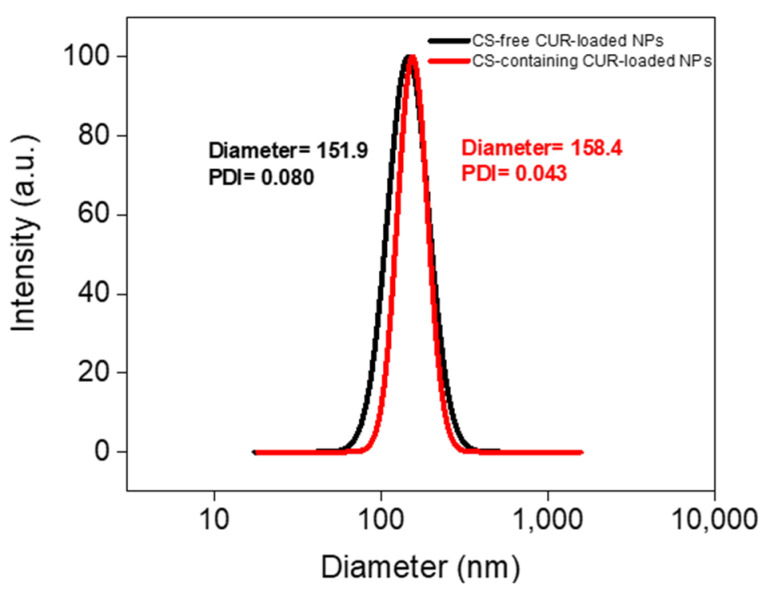
The particle sizes and size distribution of CS-free and CS-containing CUR-loaded NPs.

To examine the effects of CS on the formation of CUR-loaded NPs, the structures of CS-free and CS-containing CUR-loaded NPs were analyzed and compared. As shown in Figure 1 and Figure S1, it can be seen that as-prepared CS-free CUR-loaded NPs showed uniform and spherical structures with a diameter of 150 ± 2 nm. In the presence of CS, CUR-loaded NPs displayed an obvious core-shell structure with a diameter of 160 ± 2 nm, indicating that the CS was successfully joined into the particle and induced “phase separation” (Figure 1a,c). The stabilizer PVP was mainly concentrated in the outermost layer of the CS-containing CUR-loaded NPs, contributing a thicker protective layer (Figure 1c). However, in the absence of CS, the CUR molecules were evenly distributed in the protective layer, which resulted in the uniform structure of the NPs (Figure 1b,d). As shown in Figure 2, the hydrodynamic diameters of the different CUR-loaded NPs were 152 and 158 nm, respectively, which were in good agreement with those measured by TEM images.

**Figure 3:**
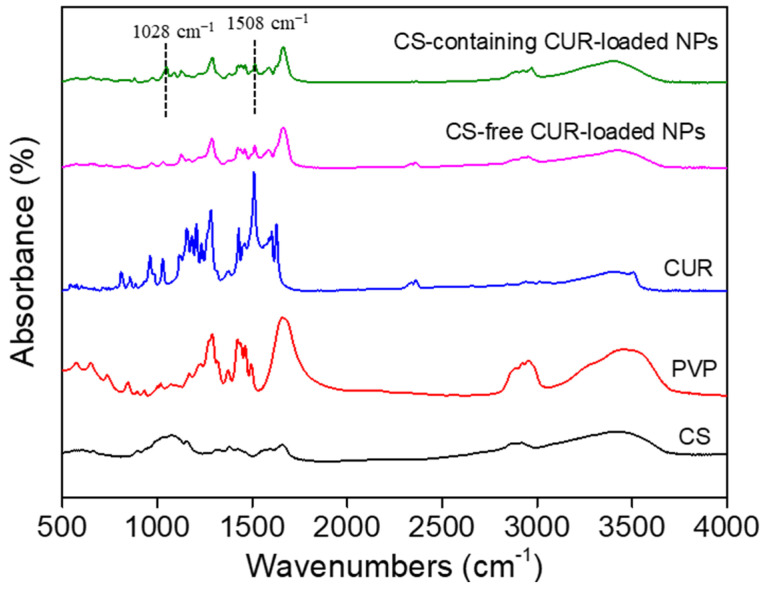
FTIR spectrum of pure CUR, CS, poly(vinyl pyrrolidone) (PVP), and CUR-loaded NPs.

As shown in Figure 3, the characteristic band of PVP observed at 2954 cm^−1^ was contributed by the vibration absorption of the asymmetrical stretching of pyrrole ring (CH_2_), and the one at 3448 cm^−1^ was attributed to the O-H stretching vibration of stronger hydrogen bond [28]. Moreover, the typical bands at 1508 and 1028 cm^−1^ CUR indicated the N-H and C=O stretching vibrations, which could be also detected in the CUR-loaded NPs spectrum. These results demonstrated that the CUR seems to be successfully encapsulated into the NPs.

**Figure 4:**
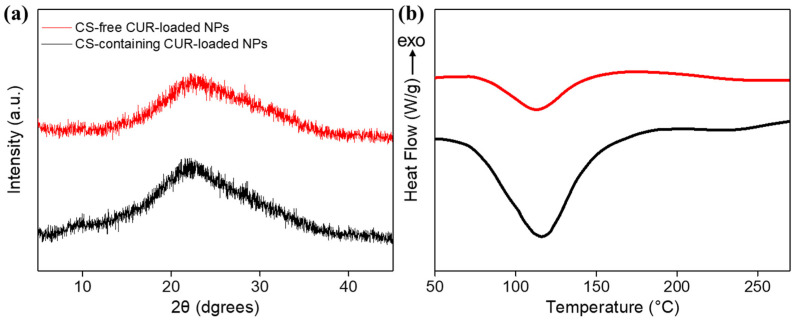
(**a**) XRD patterns and (**b**) differential scanning calorimetry (DSC) thermographs curves of CS-free (red line) and CS-containing (black line) CUR-loaded NPs.

To understand the crystal structure of CUR inside the NPs, XRD patterns of pure CUR and CUR-loaded NPs were measured in the 2θ range of 5–45° (Figure 4 and Figure S2). The crystalline CUR exhibited the sharp and intense characteristic peaks at 12° and 29° [29]. If the CUR crystals appear inside the CUR-loaded NPs, the release process of CUR will be limited. As expected, the characteristic peaks of CUR were not observed in the XRD patterns of the CUR-loaded NPs (Figure 4a), which confirmed that the CUR were loaded inside the NPs as the amorphous form [30].

In the DSC thermograms (Figure 4b and Figure S3), the pure CUR had a sharp endothermic peak at around 180 °C, which was attributed to the endothermic melting of the CUR crystal [31]. Figure S2 shows that the CS displayed an endothermic peak at around 125 °C, which can be associated with the degradation of the polymer and the losing of bound water due to the destruction of hydrogen bonds [32]. As an amorphous polymer, PVP has no fixed melting point, and an endothermic peak at around 130 °C seems to be caused by its glass transition temperature (Tg) [33]. For CS-free and CS-containing CUR-loaded NPs, melting peak of CUR crystals had not found, which further supported that the CUR was likely to be an amorphous state inside the NPs [34]. As shown in Figure 4b, the higher heat flow absorption at around 125 °C of the CS-containing CUR-loaded NPs compared to that of CS-free CUR-loaded NPs confirmed that the CS was successfully encapsulated within NPs.

### 3.2. Encapsulation Efficiency (EE) and Drug Loading Capacity (DLC)

**Figure 5:**
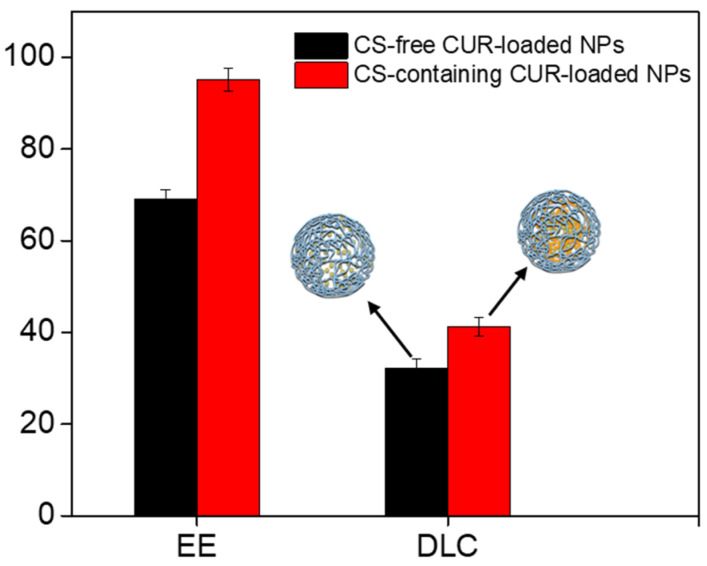
Encapsulation efficiency (EE) and loading capacity (DLC) of CUR-loaded NPs with and without CS.

As shown in Figure 5, the EE and DLC of CS-free CUR-loaded NPs were 69.1 ± 2.3% and 32.3 ± 1.9%, respectively. Interestingly, after adding natural polymer CS into the preparation process, the EE and DLC of CUR-loaded NPs could reach up to 95.2 ± 1.4% and 41.3 ± 2.1%, respectively. Probably the polyhydroxy and amino-rich structure of CS endows it with an ability to bind CUR via hydrophobic effects and inter-hydrogen bonding interaction, indicating that CUR can be effectively encapsulated in the NPs with more payloads [35,36]. In addition, the hydrophobicity of CUR could be increased due to the combination of CS and CUR during the FNP process, and thus benefiting for enhancement of the drug loading capacity [16,36,37].

### 3.3. Effect of Reynolds Number on Particle Size

**Figure 6:**
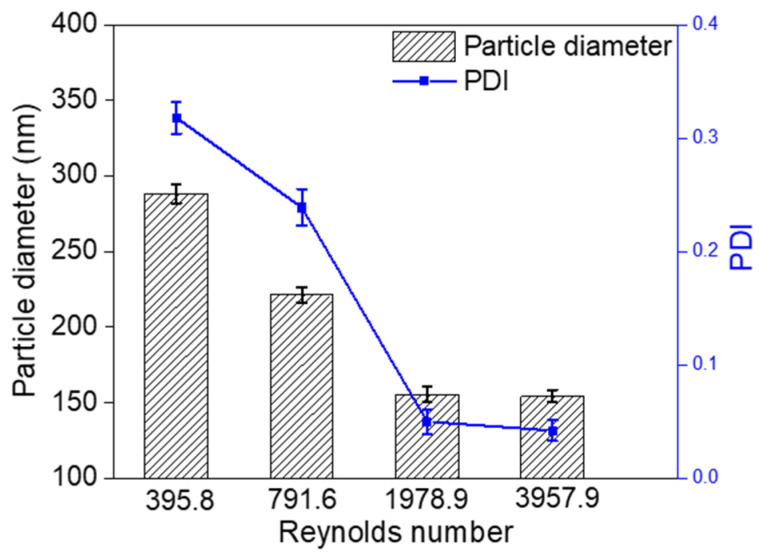
Effect of the Reynolds number (Re) on the average particle size and polydispersity index (PDI) of CS-containing CUR-loaded NPs.

In the FNP process, the mixing parameter, expressed by Re, can be flexibly controlled to adjust the size of the NPs [38,39]. As shown in Figure 6, simply changing the Re number allows preparation of CUR-loaded NPs with tunable particle sizes from ca. 160 to 290 nm. This phenomenon could be explained by the fact that the higher Re number leads to the more homogenous and effective mixing, which could lead to the smaller particle sizes. It was noteworthy that no further decrease of particle sizes was observed when the Re number reaches a certain value (Re ≥ 2000). This is probably because when the Re number is over the turbulent mixing value [27], the homogenous mixing condition is accomplished, and the size of NPs is no longer controlled by dynamics [39,40].

### 3.4. Stability of the CUR-loaded NPs

**Figure 7:**
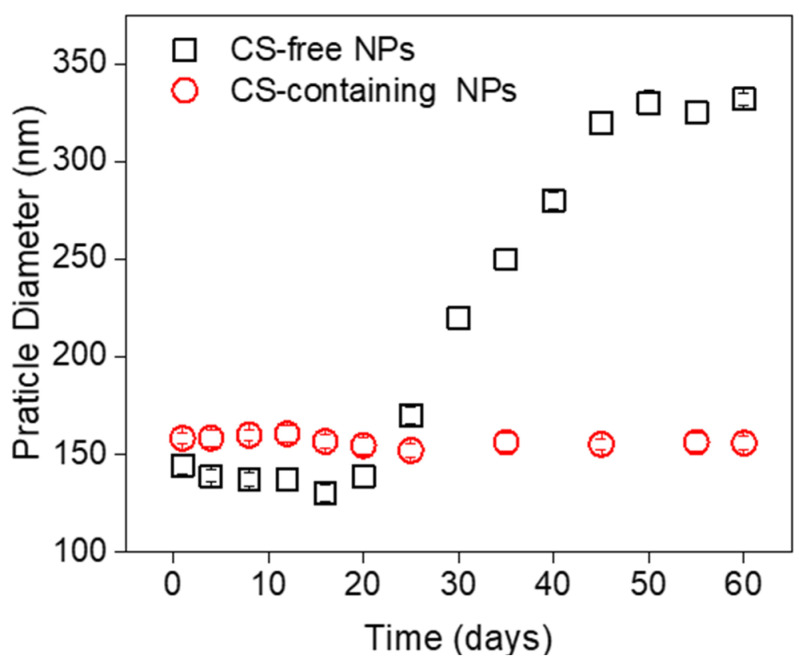
Effect of addition of CS on particle stability for CUR-loaded NPs.

The size and encapsulation efficiency of CUR-loaded NPs were monitored at defined time intervals. As can be seen from Figure 7, the size of CUR-loaded NPs prepared without using CS gradually increased from ca. 150 to 400 nm after two months, which is likely due to the Ostwald ripening phenomenon of the NPs [41]. Notably, as-prepared CS-containing CUR-loaded NPs showed excellent stability with no obvious changes in particle size over two months as compared to that of CS-free CUR-loaded NPs. The reason may be that CUR molecules inside the NPs could combine with CS through hydrogen bonding, significantly slowing down the release of the CUR and thus the Ostwald ripening [37]. The measured zeta potential of the CS-free and CS-containing CUR-loaded NPs are −15.5 ± 0.4 mV and −34.3 ± 0.5 mV, respectively, which can contribute to the stability of the NPs.

**Figure 8:**
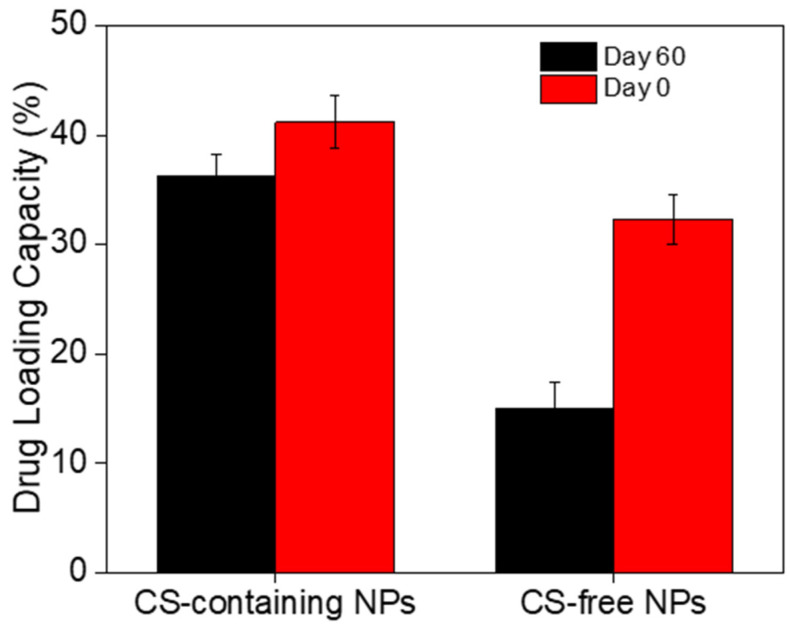
CUR loading capacity of CUR-loaded NPs before and after storage at 25 °C for 2 months.

Figure 8 shows the encapsulation efficiency of the two kinds of CUR-loaded NPs stored at room temperature for two months. A significant decrease in loading capacity of CS-free CUR-loaded NPs from 32.0 ± 2.4% to 15.0 ± 2.3% was observed, while the CS-containing CUR-loaded NPs remained quite stable with only 5.0% loss in the drug loading capacity in two months. These results demonstrate the good stability of CS-containing CUR-loaded NPs and suggest that the natural polymer CS could efficiently prohibit the leakage of drugs during the long-term storage.

### 3.5. In Vitro Release

**Figure 9:**
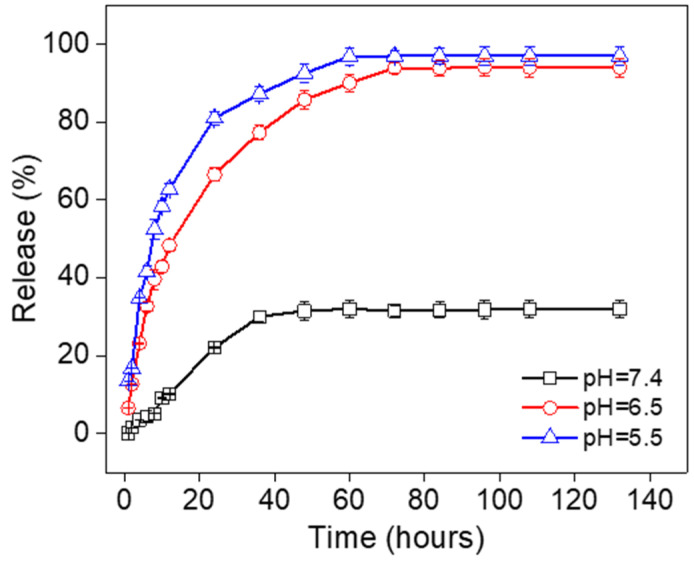
Accumulative release profiles of CS-containing CUR-loaded NPs in PBS at 37 °C with different pH values.

The release behavior of CUR from NPs were tested at various pH values (pH= 7.4, 6.5, and 5.5) in phosphate buffer solution. As shown in Figure 9, CUR exhibited an excellent pH-responsive release process from NPs, which could be related to the protonation of amino groups in CS and the reduction of the hydrogen bonding [42]. At the neutral condition (pH = 7.4), the cumulative release of drug was as low as 32.0 ± 2.4%, suggesting that CS-containing CUR-loaded NPs possessed a good stability in the regular physiological condition. However, the CUR-loaded NPs showed a fast release behavior of the drug at pH 6.5 and pH 5.5, with a cumulative release as high as 85.8 ± 2.1% and 92.5 ± 1.7%, respectively, after 48 h. These results indicated that the pH-responsive CUR-loaded NPs could effectively control the release of CUR in an acidic environment, which would be of great benefit for drug delivery.

## 4. Conclusions

In this study, a cost-effective drug delivery system with high drug loading capacity was developed based on the FNP technique. A CS-free CUR-loaded NPs with a drug encapsulation efficiency of 69.1% was rapidly prepared by FNP and showed a 20 days stability in storage. Benefiting from the excellent ability of natural polymer CS to capture CUR molecules through intermolecular forces, the encapsulation efficiency (≈95.0%) and storage stability (at least 2 months) of CS-containing CUR-loaded NPs were remarkably improved compared to that of the CS-free CUR-loaded NPs. Additionally, the particle size of the as-obtained CUR-loaded NPs could be easily tuned by controlling the mixing parameters during the FNP process. Furthermore, the loaded CUR showed an effectively targeted release with high cumulative release of 97% under the acidic environment. We envision that the use of natural polymer CS not only prepares the highly cost-effective CUR-loaded NPs with high drug loading capacity, but also endows CUR-loaded NPs with pH-responsive drug release ability for targeted drug delivery. Therefore, this work suggests that the as-prepared CS-containing nano-sized drug-loaded particles would be a very promising drug delivery system in the medical application.

## Data Availability

The data presented in this study are available on request from the corresponding author.

## Supplementary Materials

The following are available online at https://www.mdpi.com/2079-4991/11/3/734/s1, Figure S1: The overview TEM images of (a) CS-containing CUR-loaded NPs, and (b) CS-free CUR-loaded NPs. Figure S2: XRD patterns of CS, CUR, and PVP; Figure S3: DSC thermographs curves CS, CUR, and PVP.
