# Supplementary material for: A Cost-Effective Nano-Sized Curcumin Delivery System with High Drug Loading Capacity Prepared via Flash Nanoprecipitation
Source: Nanomaterials (Basel). 2021 Mar 15;11(3):734. doi: 10.3390/nano11030734 (PMC8001153; doi:10.3390/nano11030734)
Supplement: Supplementary file 1 [file nanomaterials-11-00734-s001.pdf]

nanomaterials

Supporting Information

A Cost-effective Nano-Sized Curcumin Delivery System with High Drug Loading Capacity Prepared via Flash Nanoprecipitation

Zhuo Chen [1], Zhinan Fu [1,*], Li Li [1,*], Enguang Ma [2] and Xuhong Guo [2,*]

1 State Key Laboratory of Chemical Engineering, East China University of Science and Technology, Shanghai 200237, China; chen9635@163.com
2 Engineering Research Center of Materials Chemical Engineering of Xinjiang Bingtuan, Shihezi University, Shihezi 832000, China; maenguang@163.com
* Correspondence: 10120549@mail.ecust.edu.cn (Z.F.); lili76131@ecust.edu.cn (L.L.); guoxuhong@ecust.edu.cn (X.G.)

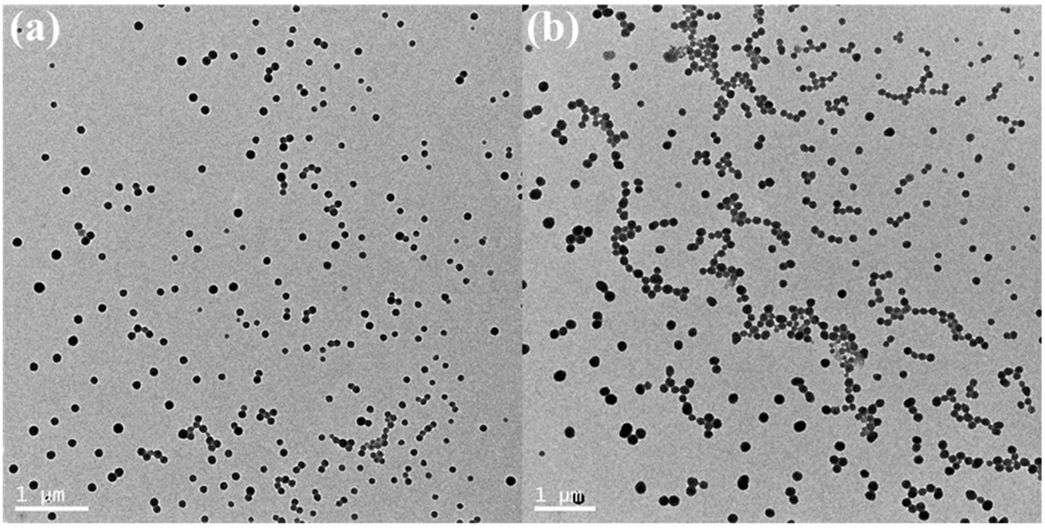

Figure S1. The overview TEM images of (**a**) CS-containing CUR-loaded NPs, and (**b**) CS-free CUR-loaded NPs.

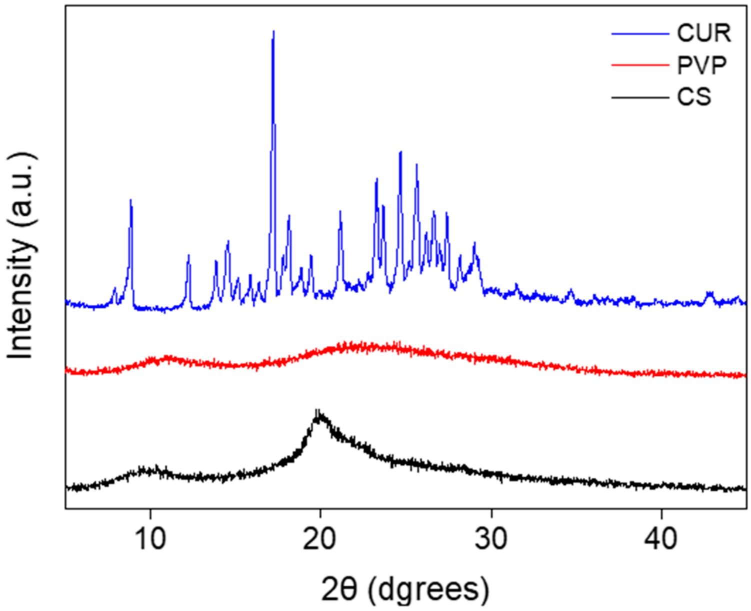

Figure S2. XRD patterns of CS, CUR, and PVP.

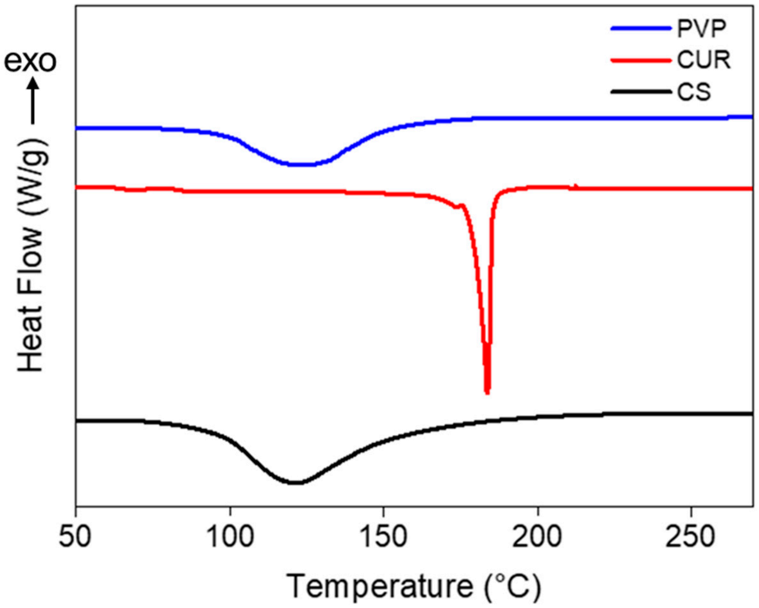

Figure S3. DSC thermographs curves of CS, CUR, and PVP.
